# How reproducible is self-reported information on exposure to smoking, drinking, and dietary patterns? Evidence among Brazilian adults in the Pró-Saúde Study

**DOI:** 10.1590/S1516-31802003000200006

**Published:** 2003-03-05

**Authors:** Dóra Chor, Eduardo Faerstein, Márcia Guimarães Mello Alves, Claudia de Souza Lopes

**Keywords:** Brazil, Smoking, Alcohol, Drinking, Diet, Brasil, Fumo, Bebida, Alcoólica, Dieta

## Abstract

**CONTEXT:**

Epidemiological studies of the validity and reliability of self-reported information on important risk factors for non-communicable chronic diseases are scarce in Brazil.

**OBJECTIVE:**

We evaluated the test-retest reliability of information – overall and stratified by gender, age and education – on active and passive smoking, alcohol intake and aspects of dietary habits.

**TYPE OF STUDY:**

Test-retest reliability.

**SETTING:**

Universidade do Estado do Rio de Janeiro, Rio de Janeiro, Brazil.

**PARTICIPANTS:**

192 University employees.

**PROCEDURES:**

Self-administered questionnaires were completed on two occasions, two weeks apart.

**MAIN MEASUREMENTS:**

Kappa Statistics; Intraclass Correlation Coefficient.

**RESULTS:**

Information on smoking status and pack-years smoked had almost perfect levels of agreement, respectively, kappa = 0.97 (95% CI, 0.92-1.00), and intraclass correlation coefficient = 0.93 (CI 95%, 0.89-0.96). Characteristics of alcohol intake yielded substantial levels of agreement (kappa ranging from 0.62 to 0.69). The reproducibility of the information on dietary habits varied from 0.67 to 0.79 (kappa). No clear-cut patterns could be identified comparing information by age or gender. There was a slight tendency towards greater reliability among people with higher levels of education.

**CONCLUSION:**

The reproducibility of information on smoking, drinking, and dietary patterns ranged from substantial to excellent, as investigated in the Pró-Saúde Study, a longitudinal investigation recently launched in Rio de Janeiro.

## RESUMO

**CONTEXTO:**

Estudos epidemiológicos sobre a validade e confiabilidade de informações auto-referidas sobre fatores de risco para doenças crônicas não-transmissíveis são escassos no Brasil.

**OBJETIVO:**

Avaliar a confiabilidade teste-reteste da informação ¾ total e estratificada por gênero, idade e educação ¾ sobre fumo ativo e passivo, consumo de álcool e aspectos de hábitos dietéticos.

**LOCAL:**

Universidade do Estado do Rio de Janeiro, Rio de Janeiro, Brasil.

**PARTICIPANTES:**

192 funcionários técnicoadministrativos.

**PROCEDIMENTOS:**

Questionários auto-administrados foram completados em duas ocasiões, com duas semanas de intervalo.

**ANÁLISE ESTATÍSTICA:**

Estatística Kappa; Coeficiente de Correlação Intraclasse.

**RESULTADOS:**

Estatística. As informações sobre o hábito de fumar cigarros e a quantidade de cigarros (maços-ano) apresentaram níveis quase perfeitos de concordância, respectivamente, kappa = 0,97 (IC 95%, 0,92 – 1,00); coeficiente de correlação intraclasse = 0,93 (IC 95%, 0,89 – 0,96). Características de consumo de álcool apresentaram níveis substanciais de concordância (kappa variando entre 0,62 e 0,69). A confiabilidade da informação sobre hábitos dietéticos variou entre 0,67 e 0,69 (kappa). Nenhum padrão definido pode ser identificado comparando a informação segundo idade ou gênero. Houve uma leve tendência à maior confiabilidade entre pessoas com maiores níveis de escolaridade.

**CONCLUSÃO:**

A confiabilidade das informações sobre o hábito de fumar, consumo de bebidas alcoólicas e padrões de dieta variou entre muito boa a excelente, de acordo com a investigação realizada no Estudo Pró-Saúde, investigação longitudinal, recentemente iniciada no Rio de Janeiro.

## INTRODUCTION

There are no published reports of epidemiological studies among Brazilian adults regarding the validity and reliability of self-reported information on smoking, exposure to passive smoking, and alcohol intake, which are important risk factors for non-communicable chronic diseases. A single Brazilian study assessing the quality of information regarding habitual diet has been made.^1^

In addition, no information exists about how the quality of self-reported information might vary across population subgroups (defined, for example, by gender, age, or socioeconomic status). Research conducted in the United States, however, has shown that information quality may vary with these and other characteristics of the study population,^2-4^ leading to measurement errors that may in part explain discrepant results between epidemio-logical studies.^5^

Information on health-related behavior, generally obtained by means of interviews or self-administered questionnaires, may be distorted by respondents’ recall errors, or by their wish to give responses which would be considered socially desirable.^6,7^ Thus, the evaluation of the quality of information obtained from epidemiological research is crucial, and the estimation of validity and reproducibility indicators is the main tool for conducting such evaluations.^8^

This paper reports on the test-retest reliability of information on smoking, exposure to passive smoking, alcohol intake, and aspects of diet (fruit and vegetable intake). It also presents stratum-specific estimates by gender, age and educational attainment. Questions on these and other exposures were subsequently included in a multidimensional, self-administered questionnaire applied to 4,030 university employees in Rio de Janeiro, Brazil. This was the Pró-Saúde Study, launched in 1999 with the objective of investigating relationships between social factors and health outcomes.

### Study population

The test-retest study was conducted in June 1999. The eligible participants were 1,120 employees who were at that time working at the University where the Pró-Saúde Study was subsequently launched, but who were not permanent employees (i.e. they were not on the administrative "tenure track" that exists in Brazil), although they were not short-term "temporary" workers either. Their job activities were similar to those performed by permanent employees, but they were considered ineligible for the main study because the prospects for their long-term follow-up were uncertain. A 20% systematic sample of 224 potential respondents (e.g. the 5^th^, 10^th^, etc.) was drawn from official listings.

### Test-retest reliability study

The conditions under which the questionnaire was administered were identical to those of the main study, i.e. during working hours, in University classrooms, and with help available from trained personnel. Among 224 employees sampled, 202 (90.2%) agreed to fill out the questionnaire. After its completion, they were invited to repeat the procedure two weeks later, with the explanation given being that the purpose was to test the adequacy of the questionnaire, rather than the participants’ responses. The procedure was repeated by 192 respondents (95.0% of the initial participants), without relevant variations in the proportions of participants in the test and retest according to age, gender, or schooling.

Responses to the following closed-ended questions are analyzed in the present paper: "How often do you eat fresh fruit?"; "How often do you eat vegetables?"; "Have you ever been a cigarette smoker, that is, have you smoked at least 100 cigarettes (five packs) during your lifetime?"; "Do you currently smoke cigarettes?"; "In general, how many cigarettes per day did you smoke or do you currently smoke?"; "In total, for how many years did you smoke or have you been smoking? (do not include time periods when you did not smoke)". The following open-ended questions were also analyzed: "Are there other people who smoke at your workplace or at home?"; "In the last two weeks, did you drink any alcoholic beverage?"; "In the last two weeks, on how many days did you drink any alcoholic beverage?"; "In the last two weeks, on average, how many drinks did you have on each of those days when you drank?"

### Statistical analysis

Agreement was measured for the entire sample, and also stratifying for gender, age (18-39 years vs. 40 years or more), and education (high school or less vs. college or more). The responses were analyzed in terms of assessing smoking status (current, former, or never); exposure to passive smoking (at workplace, at home, both or neither); alcohol intake during previous 2 weeks (yes or no), number of days of drinking (1, 2-5, or more than 5), and average number of drink units (1, 2-4, 5-7, or more than 7); usual frequency of fruit and vegetable intake (less than once a week, 1-3 days/week, 4-6 days/week, or daily). Pack-years of cigarettes smoked were calculated by multiplying the average number of cigarettes smoked daily by the duration in years of the smoking habit.

We compared frequencies reported at the test and retest by using kappa statistics (k) for categorical variables (active and passive smoking, and alcohol intake) and weighted kappa (k_w_ – squared error weighting)^9^ for ordinal variables (frequency and amount of alcohol intake; fruit intake and vegetable intake).^10^ The intraclass correlation coefficient (ICC) was utilized to measure agreement for the continuous variable pack-years smoked.^11^ According to criteria from Landis and Koch,^12^ kappa values greater than 0.80 represent "almost perfect" agreement; those between 0.60 and 0.80 show "substantial" agreement; those between 0.40 and 0.60 show "moderate" agreement, and values below 0.40 represent "poor to fair" agreement.

The independent associations of gender, age, and education with test-retest agreement (outcome variable: "agree versus disagree") were investigated by fitting logistic regression models for each health-related behavior or exposure.

## RESULTS

Overall, no substantial differences were observed between test and retest prevalence (Table 1, previous page). Information on smoking status and pack-years smoked was shown to be highly reliable: k = 0.97 (95% confidence interval, CI: 0.92-1.00) and ICC = 0.92 (95% CI: 0.88-0.95), respectively. A high level of agreement was also estimated regarding passive smoking (k = 0.73). Nevertheless, it is worth noting that the prevalence of passive smoking "at home only" estimated in the test (7.4%) was nearly double that of the retest (4.2%), which did not occur with passive smoking "at workplace only" or "at home and workplace".

**Table 1:** Test-retest agreement of self-reported information on active and passive smoking, patterns of alcohol intake and diet. Pró-Saúde Study, Rio de Janeiro, 1999

Characteristic	Test		Retest		Kappa (95% CI)
**Cigarette Smoking**	n	%	n	%	
Current	*44*	*24.9*	*46*	*26.1*	*0.97 (0.92 – 1.00)*
*Former*	35	19.8	31	17.6	
**Pack-years smoked**	12.5 (15.1)^1^		13.3 (15.3)^1^		0.92 (0.88 – 0.95)*
**Passive smoking**					
At home and at workplace	42	22.2	47	24.5	0.73 (0.65 – 0.81)
At workplace only	72	38.1	75	39.1	
At home only	14	7.4	8	4.2	
**Any alcohol intake** 2	125	67.9	113	60.4	0.69 (0.58 – 0.80)
**Frequency of alcohol intake** 2					
> 5 days	16	12.7	18	16.1	0.62 (0.48 – 0.76)**
2-5 days	63	50.0	57	50.9	
1 day	47	37.3	37	33.0	
**Units of alcohol intake** 3					
*> 7*	20	16.1	10	8.9	0.66 (0.54 – 0.78)**
*5-7*	16	12.9	23	20.5	
2-4	57	46.0	56	50.0	
1	31	25.0	23	20.5	
**Fruit Intake** ^4^					
Less than once a week	40	20.8	34	17.7	0.79 (0.73 – 0.85)**
1-3 times per week	61	31.8	78	40.6	
4-6 times per week	23	12.0	28	14.6	
Daily	68	35.4	52	27.1	
**Vegetable Intake** ^4^					
Less than once a week	21	10.9	23	12.0	0.67 (0.58 – 0.76)**
1-3 times per week	55	28.7	72	37.7	
4-6 times per week	43	22.4	33	17.3	
Daily	73	38.0	63	33.0	

1 Number in brackets = standard deviation;

2 In previous two weeks;

3 Average intake on days when drank in last two weeks (number of drinks);

4 Usual intake;

* Intraclass Correlation Coefficient;

** Weighted kappa; CI = confidence interval.

Reliability figures for characteristics of alcohol intake were lower, with the kappa statistic varying between 0.62 (95% CI: 0.48-0.76) and 0.69 (95% CI: 0.58-0.80).

Among diet-related characteristics, information on fruit intake showed the highest agreement (k_w_ = 0.79), while information on intake of vegetables ranked lower (k_w_ = 0.67).

No clear-cut patterns could be identified by comparing kappa statistics by gender and age strata (Table 2). However, it is worth noting that information on pack-years smoked showed lower reliability among younger employees. In addition, there was a slight tendency towards higher reliability among people with higher levels of education.

**Table 2:** Test-retest agreement of self-reported information on active and passive smoking, patterns of alcohol intake and diet, by gender, age and education. Pró-Saúde Study, Rio de Janeiro, 1999

Strata	Gender		Age (years)		Education	
Characteristics	Men	Women	18 – 39	40 or more	High school or less	College or more
**Cigarette smoking**						
Current	1.00 ( - )	0.94 (0.83 – 1.00)	1.00 ( - )	0.95 (0.86 – 1.00)	0.95 (0.84 – 1.00)	1.00 ( - )
Former	0.98 ( 0.93 – 1.00)	0.95 (0.89 – 1.00)	0.96 (0.90 – 1.00)	0.97 (0.92 – 1.00)	0.98 (0.93 – 1.00)	0.95 (0.88 – 1.00)
**Pack-years smoked** *	0.95 (0.90 – 0.97)	0.74 (0.55 – 0.86)	0.61 (0.35 – 0.79)	0.94 (0.90 – 0.97)	0.89 (0.80 – 0.94)	0.95 (0.90 – 0.98)
**Passive smoking**						
Home and workplace	0.64 (0.43 – 0.85)	0.85 (0.73 – 0.97)	0.81 (0.67 – 0.95)	0.71 (0.54 – 0.88)	0.74 (0.60 – 0.88)	0.75 (0.54 – 0.96)
At workplace only	0.60 (0.43 – 0.77)	0.89 (0.80 – 0.98)	0.78 (0.66 – 0.90)	0.67 (0.51 – 0.85)	0.67 (0.52 – 0.82)	0.83 (0.71 – 0.95)
At home only	0.47 (0.11 – 0.83)	0.58 (0.22 – 0.94)	0.70 (0.42 – 0.98)	0.22 (0 – 0.63)	0.42 (0.01 – 0.83)	0.59 (0.26 – 0.92)
**Any alcohol intake** 1	0.69 (0.52 – 0.86)	0.68 (0.53 – 0.83)	0.59 (0.42 – 0.78)	0.78 (0.64 – 0.92)	0.69 (0.54 – 0.84)	0.68 (0.50 – 0.86)
**Frequency of alcohol intake** 1 **	0.64 (0.44 – 0.84)	0.57 (0.35 – 0.79)	0.66 (0.51 – 0.81)	0.57 (0.32 – 0.82)	0.56 (0.34 – 0.78)	0.71 (0.55 – 0.87)
**Units of alcohol intake** 2 **	0.66 (0.51 – 0.81)	0.64 (0.45 – 0.83)	0.65 (0.50 – 0.80)	0.68 (0.53 – 0.83)	0.64 (0.46 – 0.82)	0.64 (0.47 – 0.81)
**Fruit intake** 3 **	0.80 (0.71 – 0.89)	0.78 (0.69 – 0.87)	0.71 (0.60 – 0.82)	0.84 (0.77 – 0.91)	0.75 (0.65 – 0.85)	0.83 (0.75 – 0.91)
**Vegetable intake** 3 **	0.73 (0.63 – 0.83)	0.60 (0.63 – 0.83)	0.67 (0.55 – 0.79)	0.66 (0.52 – 0.80)	0.65 (0.51 – 0.79)	0.69 (0.57 – 0.81)

1 *In previous two weeks;*

2 *Average intake on days when drank in last two weeks (number of drinks);*

3 *Usual intake;*

* *Intraclass Correlation Coefficient;*

** *Weighted kappa*

The association between agreement and potential sociodemographic predictors (gender, age, and education) was further investigated -except for smoking, as this yielded reliability values close to 1.0 (data not presented). The responses regarding number of alcohol units, in-take of vegetables and fruit showed no association (p < 0.10) with any of these potential sociodemographic predictors. However, women were over three times more likely than men to agree on passive smoking information: crude odds ratio, OR = 3.3; (95% CI: 1.5-7.2); after adjusting for age and education, OR = 3.6 (95% CI: 1.6-8.3) (data not shown in table).

## DISCUSSION

Our results indicate good levels of reliability regarding information on important risk factors for major adult chronic diseases in this Brazilian study population. Reliability of the information on smoking, number of cigarettes smoked and exposure to passive smoking (except at home) ranged from substantial to almost perfect. Reproducibility of the other items regarding dietary patterns and alcohol intake ranged from moderate to almost perfect.

Studies conducted in the United States have also considered information on tobacco use and alcohol intake to be reasonably reproducible.^13,14^ The reproducibility of information on dietary patterns tends to show more variability depending on the data collection instrument utilized and the frequencies of in-take of specific dietary items.^2,4^

The estimated measures of reliability have practical implications for future analyses of the Pró-Saúde Study data. We were especially interested in assessing possible differences across the responses given by sociodemographic subgroups. Gender, age, and education were each predictive of some variation in agreement for one or more risk factors, but no consistent effect of any of these characteristics on the reproducibility of information was observed. Therefore, the overall similar prevalence of the risk factors measured in both the test and the retest, and the absence of systematic differences in the information across sociodemographic strata suggest that potential misclassification of results in the main study would result mostly from non-differential errors.

Stein et al.^3^ found similar results when investigating demographic predictors of agreement in information on chronic disease risk factors: there was no consistent effect of any demographic characteristic across risk factors, although some of them predicted higher levels of concordance for one or more risk factors. In contrast, in two other studies,^2,15^ agreement on information about preventive health practices and physical activity variables tended to be lower among non-white than white women.

Although good reliability does not ensure validity, reliability studies are often conducted insofar as they provide information on consistency and stability of the answers.^2-4,13-16^ One important limitation of the test-retest method in estimating reproducibility must be considered. Any actual change in the condition or exposure under study, which may occur during the period between the test and the retest, will inevitably result in underestimated reliability. This may have been the case with the moderate reliability estimated in this study for the information on alcohol intake during the previous two weeks.

## CONCLUSION

The need for reliability studies built into epidemiological investigations should not be overlooked, as levels of reliability can vary according to characteristics of the population and data collection methods. The reproducibility of information on smoking, drinking, and dietary patterns ranged from substantial to excellent, as investigated in the Pró-Saúde Study, a longitudinal investigation recently launched in Rio de Janeiro.
